# Vaginoplasty for Disorders of Sex Development

**DOI:** 10.3389/fendo.2013.00029

**Published:** 2013-03-11

**Authors:** Nino Guarino, Salvatore Scommegna, Silvia Majore, Anna Maria Rapone, Luciana Ungaro, Aldo Morrone, Paola Grammatico, Giacinto A. Marrocco

**Affiliations:** ^1^Department of Pediatric Surgery, S. Camillo-Forlanini HospitalRome, Italy; ^2^Department of Pediatrics and Hematology, S. Camillo-Forlanini HospitalRome, Italy; ^3^Department of Medical Genetics, Molecular Department, University “La Sapienza”Rome, Italy; ^4^Psychology Department, S. Camillo-Forlanini HospitalRome, Italy; ^5^Healthcare Administration, S. Camillo-Forlanini HospitalRome, Italy

**Keywords:** vaginoplasty, DSD, intersex, genitoplasty, neovagina

## Abstract

One of the most common problem found in patients with Disorders of Sexual Developments is the absence or extreme hypoplasia of the vagina. The type of patients presenting this anomaly may belong to completely different groups: (1) Patients with a urogenital sinus with urethra and vagina fusing together to form a common channel. (2) Patients with absent Müllerian structures and different degrees of external virilization. (3) Complex malformations. Treatment options: treatment of these patients is under discussion and may consist, basically, in non-operative dilation methods or surgical creation of a neovagina. Consensus is far to be reached among the various surgical subspecialties regarding the optimal method of vaginal replacement. Adequate number of long-term follow up patients are still non-available so that most conclusions are based on small number series. The authors describe the different treatment options in detail.

The concept of surgery for Disorders of Sexual Developments (DSD) conditions has become increasingly controversial in the last decade. Clinicians and patients have become involved in the debate, with strong views on both sides of the fence. The management of DSD conditions poses a huge challenge for the clinician. Almost every aspect of DSD is controversial but by far the most difficult and confusing area is that of surgery.

The primary goals of feminizing genital reconstruction are to create a normal-looking, sensate clitoris, provide an adequately sized and appropriately situated vagina, and create normal-appearing female external genitalia. In the long-term the ultimate goals of feminizing genital reconstruction are to provide good cosmetic results, potential for satisfactory vaginal intercourse, and if possible fertility (Hoepffner et al., 2006).

One of the most common problem found in patients with DSD is the absence or the extreme hypoplasia of the vagina.

The type of patients presenting this anomaly may belong to one of the following groups:

1. 46XX DSD: individuals with inappropriate secretion of testosterone during intrauterine life causing the formation of a urogenital sinus where urethra and vagina fuse together to form a common channel. To this group belongs one of the most common condition leading to DSD: congenital adrenal hyperplasia (CAH). The external genitalia are more or less virilized correlating with a more or less high confluence of the vagina into the urogenital sinus.
2. 46XY DSD: individuals with absent Müllerian structures and different degrees of external virilization. The most frequent defect is complete androgen insensitivity syndrome (CAIS). In this condition the uterus together with the proximal vagina are absent while a variable portion of distal vagina exist together with normal external feminine genitalia.
3. Mayer–Rokitansky–Kuster–Hauser (MRKH) syndrome: individuals with a 46XX karyotype and failure of the Müllerian ducts to develop. The complete or partial absence of the cervix, uterus, and vagina is associated with normal ovaries and normal hormonal pattern with complete development of external feminine genitalia.
4. Cloacal anomalies and bladder exstrophy: this group of patients are extremely heterogeneous and the severity of vaginal anomalies may vary considerably, sometime as the result of surgery procedures required to correct the primary malformations that can lead to severe stenosis, scarring, or tissue disruption.

As a consequence of these different conditions vaginoplasty is performed either as part of feminizing genitoplasty in a patient with ambiguous genitalia (e.g., CAH) or as a single procedure in patients with vaginal hypoplasia or agenesis such as patients with CAIS or MRKH.

## Timing of Treatment

Before entering into the details of the different techniques for vaginoplasty it is extremely important to briefly review the different opinions regarding the ideal time for surgery. Up to date, the best timing for feminizing genital reconstruction remains controversial. Suggested timing ranges between surgery in the small infant (6–12 months) to help parents to accept the child more easily to surgery at adolescence, when the patient can provide informed consent and genitalia reach their adult anatomy.

As said above, two are the possible clinical scenario in which a decision about when a vaginoplasty has to be performed:

– Vaginoplasty as a part of feminizing genital surgery in children with virilized external genitalia.
– Vaginoplasty alone.

In the first case the hypothesized impact of ambiguous genitalia on child development and on the relationship with the parents may “force” the surgeon to undertake an early feminizing genitoplasty including at the same time a vaginoplasty in order to complete the correction as a single stage. The concept underlying the early surgical management of DSD conditions arises from the Optimal Gender Policy proposed by Money in the 1950s (Money et al., 1955). Moreover the psychological trauma for the child due to the virilized external appearance and need for correction before the age of permanent memory has been emphasized (Engert, 1989; Hrabovszky and Hutson, 2002). As said above early surgery is thought to help parental acceptance of the child’s assigned gender, and improving the psychological outcomes for the child (Crouch et al., 2004; Warne et al., 2005).

Early feminizing genitoplasty is commonly performed during the first year of life. This is despite the fact that the child will not menstruate for a further 10 or so years and is unlikely to be sexually active until after puberty. Many recent studies have demonstrated very high rates of introital stenosis and frequent requirements for repeat reconstructive surgery in adolescence before tampon use or intercourse. Delaying vaginoplasty until puberty may lead to better vaginal healing when the skin is fully estrogenized. Adjunctive treatments to prevent stenosis such as vaginal dilators may be helpful and cannot be used in the young child.

## Surgical Techniques

### Urogenital sinus anomalies and disorders of sex development

#### Vaginoplasty associated to feminizing genitoplasty

The severity of the vaginal defect has historically directed the choice of procedure. The aim of surgery is to open up the lower vagina and the procedure chosen depends upon whether the confluence between urethra and vagina is high or low. Preoperative evaluation is essential to determine the type of vaginoplasty best suited to the patient. The goal of the preoperative evaluation is to understand the anatomy of the UGS. The key information needed is the length of the UGS and the specific location of the vaginal confluence in relation to the bladder neck and the UGS orifice. Genitography and endoscopy are the important preoperative studies used to define the UGS anatomy. Genitography may frequently fails to demonstrate the confluence and pre-operatory endoscopy is recommended.

##### Low confluence urogenital sinus patients

Posterior skin flap vaginoplasty is the most widely used technique. Flap vaginoplasty involves creating of an inverted U-flap (that carries Fortunoff’s name today) from the perineum and laying this into the posterior distal vagina to increase the caliber (Fortunoff et al., 1964). The posterior wall of the vagina must always be entirely incised through the small dysplastic confluence and deep into the posterior wall to prevent stenosis (Passerini-Glazel, 1999).

##### High confluence urogenital sinus patients

Historically the first reported technique for the treatment of high confluent vagina was by Hendren in 1969 and it consisted in a perineal approach locating the vagina by palpating a Fogarty balloon inserted in the UGS, incising over the balloon, and then disconnecting the vagina from the urethra. The vagina was exteriorized by laying in local skin flaps (Hendren and Crawford, 1969). The cosmetic and functional results were far from optimal leaving the patient with a perineal stenotic hole.

Passerini-Glazel (1989) described an ingenious single stage clitorovaginoplasty for the severely masculinized female. The main feature of this technique was the use of the UGS tissue for the anterior wall of the vagina. The opened UGS was combined with phallic skin flaps to create a mucocutaneous lining for the distal neovagina and introitus. The advantages of laying in the reflected UGS tissue, the lateral phallic skin flaps, and the posterior flap as individual components is to avoid a circular anastomosis that could result in stenosis (Passerini-Glazel, 1994).

More recently a new approach have become popular. A paper from Pena (1997) described an evolution of his own technique for the treatment of persistent cloaca (a complex anorectal and genitourinary malformation, in which the rectum, vagina, and urinary tract meet and fuse creating a single common channel). He simplified his original technique which initially included the separation of the three structures by avoiding to separate the urethra from the vagina, mobilizing in this way the urogenital sinus as a single unit. He named this “total urogenital mobilization” (TUM). This technique using a posterior sagittal approach and a circumferential mobilization of the entire UGS brings the UGS and vagina to a more superficial position, allowing midlevel vaginal confluences to easily reach the perineum without vaginal separation. Because this approach completely mobilizes the posterior vaginal wall, it greatly facilitates the placement of a deep posterior skin flap.

Further advancement in this technique have been introduced by Richard Rink who emphasized the use of the excess of mobilized sinus tissue by splitting it longitudinally for use as a mucosa-lined vestibule or to complete the vaginoplasty, eliminating in this case the need for a Fortunoff skin flap and consequently reducing the risk for introital stenosis (Rink and Yerkes, 2001).

Concerns arisen using TUM technique about possible detrimental consequences due to the very proximal, circumferential dissection above the pubo-urethral ligament. This region is thought to be fundamentally important to urinary continence and clitoral sensation, and may be susceptible to damage. The long-term consequences of proximal TUM dissection on the urinary tract and sexual function have been recently questioned (Davies et al., 2005a,b). For this reason a simplified approach to UGS has been reported. In order to minimize potential long-term morbidity a limited proximal dissection may, in many cases, be sufficient to obtain sufficient mobilization. This modification has been termed the partial urogenital mobilization (PUM) (Rink et al., 2006). The PUM utilizes the surgical principles of the TUM, but the dissection of the common channel is limited distal to the pubo-urethral ligament. Moreover the initial limited dissection during PUM does not preclude, when the vagina will remain at a significant distance from the perineum, to go for further proximal dissection, and division of the pubo-urethral ligament, thereby converting to a TUM.

Once again, early surgery is not the only option, especially in complex high vaginal confluence. In non-operated patients at puberty it may be observed an increase in the size of the vagina with descent of the confluence of the vagina and urogenital sinus toward the perineum. The natural enlargement of the vagina presumably occurs in the appropriate hormonal environment at puberty, and it may also be secondary to a degree of tissue expansion induced by long-term obstruction to vaginal drainage from narrowing at the vaginal orifice (Nyirady et al., 2008).

### Vaginal hypoplasia or agenesis

#### Progressive dilation – non-operative methods

Frank (1938) proposed the creation of a neovagina by applying progressive pressure to the perineum or vaginal dimple in patients with vaginal agenesis. Since this time it has been shown to be an effective technique (Ismail-Pratt et al., 2007), although dilation treatment may take several months to achieve the final result. It involves insertion of vaginal molds of gradually increasing length and width into the vaginal dimple, applying local pressure, and increasing the potential space between rectum and bladder. Vaginal dilation has few complications, as there are no anesthetic and surgical risks. However, it is usually a time-consuming exercise taking several months to achieve an adequate vaginal length and capacity. Success is directly related to compliance, and appropriate psychological support is essential.

Ingram (1981) modified this technique based on several other deterrents to Frank’s method, including lack of patient motivation and emotional immaturity, hand and finger fatigue, the need to squat, and the inability to perform other productive activities during the hours of pressure required. Patients were instructed to sit on a bicycle seat with dilators specially designed to allow progressive dilation by the weight of the patient’s trunk (Ingram, 1981).

Although often offered as first line therapy, it seems that the ideal candidate for the dilation method must be an emotionally mature, highly motivated older (older than 18 years) patient who wishes to avoid surgery.

#### Progressive dilation – operative methods

##### The Vecchietti procedure

It was first described in 1965 (Vecchietti, 1965) and later it has been modified to a laparoscopic approach (Ismail et al., 2006). An acrylic olive is positioned within the vaginal dimple and is connected to threads that are then passed through the rectovesical space, into the peritoneal cavity under laparoscopic control and through to the abdominal wall, where they are attached to a traction device. Steady increasing traction is applied to the threads, and the neovagina is stretched by approximately 1 cm per day. The woman remains in hospital for analgesia and the traction device and olive are removed after 7–10 days. Vaginal dilation or intercourse is required to maintain the length of the vagina. Patients older than 30 years or those with a scarred perineum may not be ideal candidates for this procedure due to tissue inelasticity, while young patients may be too immature to practice the regular dilation or regular sexual intercourse required to maintain adequate results (Brun et al., 2002). This procedure is contraindicated in patients with an enlarged urethra related to attempts at intercourse by this route, vulvar hypoplasia or a centralized urethral meatus (Fedele et al., 1994).

##### Balloon vaginoplasty

This new and novel approach mimics the Vecchietti procedure. A Foley catheter introduced laparoscopically into the rectovesical space is used instead of the traction wires, while the catheter balloon is inflated progressively to stretch the vagina (El Saman, 2010). It is claimed that compared to the small and solid acrylic “olive,” balloon distension would stretch the vagina not only in length but also in width with less pain and discomfort to the patient. A neovagina depth of 8–12 cm has been reported on day 7 following the procedure (El Saman et al., 2011). Although the results seem to be interesting, only six cases have been reported so far and there is no long-term follow up data on this group of patients.

#### Procedures where a neovagina is created within the rectovesical space and lined with different types of tissue

##### The McIndoe–Reed vaginoplasty

The operative approach involves, through a perineal incision, the creation of a plane of dissection in the retrovesical space (Banister and McIndoe, 1938). A split thickness skin graft is then harvested placed around a mold made from various materials and then inserted into the created retrovesical space and secured to the labia. The mold is left *in situ* for 7–10 days initially, after which regular dilation or sexual intercourse is required in order to maintain vaginal patency. Proponents of this approach cite the advantages of no abdominal incision, however it leaves visible scars at the origin of the skin graft that are often unacceptable to young women. Vaginal dryness is a common problem but the most devastating consequence is a high rate of graft stenosis needing frequent and potentially lifelong dilation. The likelihood of stenosis is increased if the mold is removed prematurely or regular sexual activity does not occur (Klingele et al., 2003).

##### The Davydov procedure

The procedure was initially described as an open procedure (Davydov and Zhvitiashvili, 1974) but is increasingly performed laparoscopically (Giannesi et al., 2005). In this procedure, the space between rectum and bladder is developed from the perineum and abdominally under laparoscopic guidance. It is then lined by peritoneal flaps that are freed laparoscopically from the pelvis. The peritoneum is sutured circumferentially to the introitus and a soft mold is left *in situ* and removed after 1 week. The vault of the neovagina is created either by a purse-string suture or by suturing large bowel serosa at the top. The Davydov procedure is suitable for women who have had previous pelvic surgical procedures and therefore the perineum is scarred and not amenable to stretching and dilation. As with most other vaginoplasty techniques, long-term data on sexual function are not available. In a single study reporting follow up data, the majority of women after a Davydov procedure are sexually active and report good sexual function (Giannesi et al., 2005), while 24% of these patients reported poor scores which were mainly related to psychological aspects of intercourse. In a proportion of women, however, pain and decreased lubrication can be of concern.

##### Vulvovaginoplasty – creation of a perineal pouch

Initially described by Williams (1976), this technique fell into general disuse until modified by Creatsas et al. (2001). It involves a U-shaped incision, extending from the medial side of the labia at the level of the external urethral meatus, down across the perineal body to form a skin flap. The tissues are then mobilized and sutured in layers to form a pocket in the perineum to allow coitus (Deans et al., 2010). The efficacy of the original procedure has been limited to the fact that the created vagina is external, short, and unsatisfactory for penetrative intercourse. Anecdotally patients complain the vagina is too short and there is no feeling of penetration. Dilators are difficult to use due to the angle of the external vagina, the reported success of Creatsas modification is probably due to the support to the posterior fourchette allowing coital dilation to extend the vaginal length.

##### Intestinal vaginoplasty

Intestinal vaginoplasty requires a combined perineal – abdominal approach. It remains an operation with significant morbidity as it involves resection and anastomosis of bowel. The recto-vaginal space is dissected, while a loop of either colon or ileum is resected and fed through to the perineum with its vascular pedicle maintained. The caudal edge of the intestinal segment is then sutured to the perineum. Unlike the Vecchietti and Davydov procedures which are now invariably done laparoscopically, laparoscopic intestinal vaginoplasty is now beginning to gain popularity as this procedure has been more frequently done through a laparotomy (Imparato et al., 2007).

The distal sigmoid colon is most commonly used for vaginoplasty due to its location, making the operation technically easier compared with small bowel where the short mesentery at many times prevents tension free anastomosis (Lima et al., 2010). Small bowel segments may also be used for creating a neovagina with the added benefit of less mucous production, and colitis in the graft. However, small bowel segments often have thicker mesenteries, making them harder to reach the perineum without tension (Hensle and Reiley, 1998).

Overall, the main advantages of this operation are the adequate vaginal length, the lack of shrinkage, and the natural lubrication provided by the mucous production (Gatti et al., 2010). Another significant benefit of this procedure is reported to be the avoidance of post-operative vaginal dilatation. Despite all these advantages it must be kept in mind that this is a complex procedure with a complication rate up to 19–36% (Hensle et al., 2006) and therefore should be reserved only for selected cases such as complex cloacal anomalies with absent vaginal dimple. Mucus production, diversion colitis, and malignancy risk are important concerns of this procedure.

### Conclusions and future directions

Despite favorable results, vaginoplasty remains a major surgical procedure. Several different surgical options have been reported in relation to different aspects involving the surgeon, the patient, and the anomaly (surgeon preference; clinical condition; age of the patient). The surgeon personal attitude and training is an important factor in choosing for instance a gynecological procedure (McIndoe) versus a general surgical approach that requires to manipulate the bowel (intestinal vaginoplasty), the growing experience of the laparoscopic techniques will probably further increase, in the future, their use. The clinical picture itself requires specific considerations. For instance a scarred perineum preclude the use of techniques of progressive dilatation or the presence of major associated malformation will require a complex reconstructive approach.

Finally, the timing for surgery is the most controversial issue. Early surgery has been proposed in the past with the view of bringing everything “back to normality” in an early stage of physical and emotional development. This view has been very much criticized especially in condition like CAIS o MHKR where the absent vagina contrasts with normally looking external genitalia. In addition to the above reported considerations (consent of the individual, need for additional surgery at puberty, need for vaginal dilation, etc.), a recent study by Routh et al. (2010) evaluated the hypothesized reduction of quality of life (QOL) as a consequence of the congenital absence of the vagina. To justify, from a cost-effectiveness view, a very early vaginoplasty (at 2 years of age) the supposed reduction of QOL should have the same amplitude as that of other severe conditions like bilateral vision loss or moderate mental retardation, while a vaginoplasty at the age of 10 years might be justified (in term of cost-effectiveness) for a supposed reduction in QOL similar to that of a patient with renal failure receiving hemodialysis or a patient with lymphoma undergoing chemotherapy.

Furthermore, the choice for the time of treatment, in a patient with masculinized external genitalia and absent vagina, is much more complex with three main opposing but rational views. Some think that all three procedures (clitoroplasty, labioplasty, and vaginoplasty) should be done in combination when the patient is a small infant. Others think that delaying these procedures until puberty or until the child can participate in the decision for or against surgery is appropriate.

**Figure 1:**
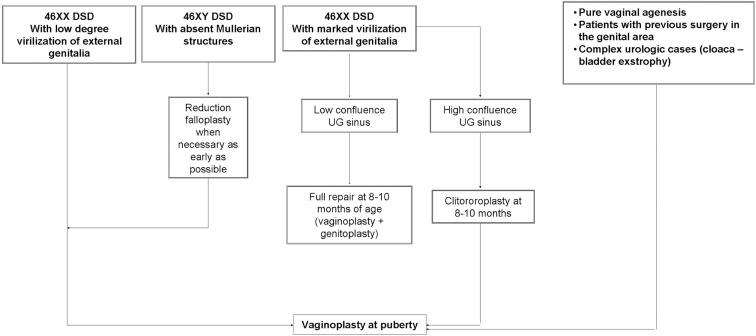
**Algorithm for vaginal replacement in childhood**.

We believe that an hybrid of these two, whereby the clitoroplasty and labioplasty are performed early and the vaginoplasty is delayed until puberty, may actually be identified as the most appropriate choice (Figure 1). This approach make possible to adapt the phenotypic appearance of external genitalia to the assigned sex and, at the same time, delay vaginoplasty after the onset of menarche allowing the subsequent hematocolpos to act as a natural tissue expander, improving the available quantity of vaginal tissue (Taghizadeh and Wilcox, 2005; Nyirady et al., 2008).

## Conflict of Interest Statement

The authors declare that the research was conducted in the absence of any commercial or financial relationships that could be construed as a potential conflict of interest.
